# Time to review policy on screening for, and managing, hypertension in South Africa: Evidence from primary care

**DOI:** 10.1371/journal.pone.0208983

**Published:** 2019-01-10

**Authors:** Margaret Thorogood, Jane Goudge, Chodziwadziwa Whiteson Kabudula, Felix Limbani, Jacqueline Roseleur, Francesc Xavier Gómez-Olivé

**Affiliations:** 1 Division of Health Sciences, University of Warwick, Coventry, United Kingdom; 2 School of Public Health, Faculty of Health Sciences, University of the Witwatersrand, Johannesburg, South Africa; 3 Centre for Health Policy, School of Public Health, Faculty of Health Sciences, University of the Witwatersrand, Johannesburg, South Africa; 4 MRC/Wits Rural Public Health and Health Transitions Research Unit (Agincourt), School of Public Health, Faculty of Health Sciences, University of the Witwatersrand, Johannesburg, South Africa; Boston University, UNITED STATES

## Abstract

**Background:**

Current policy in South Africa requires measurement of blood pressure at every visit in primary care. The number of patients regularly visiting primary care clinics for routine care is increasing rapidly, causing long queues, and unmanageable workloads.

**Methods:**

We used data collected during a randomised control trial in primary care clinics in South Africa to estimate how changes in policy might affect workloads and improve identification of undiagnosed hypertension.

**Results:**

The prevalence of raised blood pressure increased with age; 65% of individuals aged over 60 years had a raised blood pressure, and 49% of them were not on any treatment. Over three months, eight health facilities saw 8,947 individual chronic disease patients, receiving 22,323 visits from them. Of these visits, 60% were related to hypertension, with or without HIV, and a further 35% were related to HIV alone. Long waits for blood pressure checks caused friction at all levels of the clinics. Blood pressure machines frequently broke down due to heavy use, and high blood pressures readings were often ignored. If chronic disease patients without a diagnosis of hypertension had their blood pressure checked only once a year, the number of checks would be reduced by more than 80%. Individuals with hypertension had a blood pressure check on average once every 7 weeks, but South African guidelines recommend that this should be done every 3 months at most.

**Conclusions:**

The numbers of chronic disease patients in primary care clinics in South Africa is rising rapidly. New policies for measuring blood pressure in these patients attending clinics are urgently needed.

**Trial registration:**

Current Controlled Trials ISRCTN12128227 5^th^ March 2014.

## Introduction

South Africa’s health services are managing one of the largest epidemics of HIV in the world [1]. At the same time, health services are dealing with an ageing population [2] where hypertension has become a major chronic condition, affecting as much as 70% of the population aged over 40 years[3, 4]. As anti-retroviral therapy for HIV is rolled out, primary health care services are struggling to cope with a rapid increase in the number of people attending clinics regularly for anti-retroviral treatment [5, 6], as well as increasing numbers of patients visiting clinics regularly for the management of hypertension.

Despite the increasing numbers receiving care for hypertension, there is also a large proportion of the population where individuals have raised blood pressure but are not receiving any treatment [3]. This may be because the condition has not been diagnosed, or because patients have dropped out of treatment and been lost to follow up. An effective system for routine screening for hypertension could potentially address some of this problem.

Recognising the pressures of increasing demands on primary care, the South African Government has introduced a new system of managing chronic conditions in primary care, the Integrated Chronic Disease Management system [7], which aims to provide a separate service for chronic care patients, as compared with that provided for patients attending for acute care. The aim is to reduce waiting times in the clinics and improve record keeping, but this reorganisation also created extra tasks for nurses, who were already under pressure from increasing numbers of chronic disease patients.

At the time that the Integrated Chronic Disease Management system was introduced, there was no review of an existing policy on screening patients arriving at the clinics. This policy, which did not distinguish between acute and chronic clinic attendances, recommends that all patients attending clinics undergo routine health checks on every visit to “routinely check and record weight, blood pressure, pulse and temperature” [8]. In this paper, we argue that this policy is no longer appropriate for the management of regular visits from patients receiving chronic care.

The data we have used in this paper was collected during a randomised trial and its accompanying process evaluation. Between 2013 and 2015, we examined whether the introduction of trained local lay health workers to assist in the management of chronic care, and particularly the management of patients with hypertension, would relieve some of the pressure on the nurses, thus allowing them to focus more on clinical decision making and hence reduce the prevalence of untreated raised blood pressure in the community.

Four clinics in the Agincourt Health and Demographic Surveillance System site were randomised to receive the extra assistance, while four other clinics acted as controls, with no extra assistance [9]. The results of the trial were negative with no difference in management of hypertension in communities that were served by intervention clinics as compared with those served by control clinics [10]. Nevertheless, we found a significant difference in the functioning of the intervention clinics as compared to the control clinics, with higher numbers of patients with hypertension attending regularly and coming on the correct appointment day.

As part of the process evaluation alongside the randomised trial, we installed a system to collect information on all visits to the clinics for management of chronic disease, and undertook repeated observations in clinics, and interviews with all cadres of clinic staff. We observed that an important source of tension in all the clinics was the ‘vital signs queue’ where all patients who have arrived at the clinic that day have their weight, blood pressure, pulse and blood pressure measured. Moreover, we observed that the blood pressure machines often broke down and the cuffs wore out quickly, leading nurses to sometimes disregard the blood pressure measurement as unreliable [11].

In this paper, we have used quantitative data that we collected during the trial to explore the implications of the current policy for screening for hypertension in primary care clinics, and for managing diagnosed cases of hypertension. We were interested in the potential for reducing the number of checks, which would free nurses’ time to focus on identification and treatment of people with hypertension.

## Methods

We used data on current activity in the clinics to estimate the change in workloads in different possible scenarios: if patients with a diagnosis of hypertension had their blood pressure checked just four times a year, and chronic disease patients without a diagnosis of hypertension had their blood pressure checked once a year instead of every time they returned for a repeat prescription. We also used the population level data we had collected to estimate the number of people with raised blood pressure not currently on treatment but attending clinics for other chronic diseases (mainly HIV) and hence to estimate the potential yield of new diagnoses of hypertension that could be acted upon by nurses if they had more time available.

### Data sources

Table 1 shows the three sources of data that we used; data from the records kept in the clinics, data from population surveys, and data from the work of the lay health workers employed in the clinics, who had a special responsibility to check the routine blood pressure records for high readings. These data sources are described in more detail below.

**Table 1:** Summary of data used.

	Information used	Source	Type of data used	Duration of data collection	Duration used in the primary analysis in this paper
1	Clinic attendance rates by BP diagnosis	Clinical records in 8 clinics collected by data clerks	Attendance, appointment dates, recorded diagnosis	May 2014 to July 2015	Aug 2014-July 2015 (*for average annual visits and sensitivity analysis)* May 2014 –July 2015 *(for main analysis)*
2	Population prevalence of treated and untreated hypertension	Baseline and end-of-intervention independent population surveys (data combined for this paper)	BP measures, self-reported diagnosis of hypertension,	Sept–Dec 2013 and Sept–Nov 2015	Sept–Dec 2013 and Sept–Nov 2015
3	Patient journey of patients newly identified with raised BP	Records kept by lay health workers in four intervention clinics	Newly identified raised BP, subsequent clinic attendance and whether hypertension diagnosed	May 2014 –July 2015	May 2014 –July 2015

All data collected included age and sex of participants.

### Estimating levels of clinic activity from individual patient data (Table 1, row 1)

During the Nkateko trial we collected information on patients who used any of the eight primary care clinics that were participating in the trial. In each clinic, a data entry clerk equipped with a laptop collected identifying information (name, surname, national ID numbers, cell phone number, date of birth, gender, village of residence, and the name of another person in the household) from consenting patients attending for chronic disease appointments and used those data to uniquely identify each patient. The data clerk recorded every subsequent visit by a patient who had been entered into the database, and collected information from clinic records on the reason for the visit, diagnosis and treatment. No information was collected on visits by patients with acute (as opposed to chronic) conditions.

**Fig 1:**
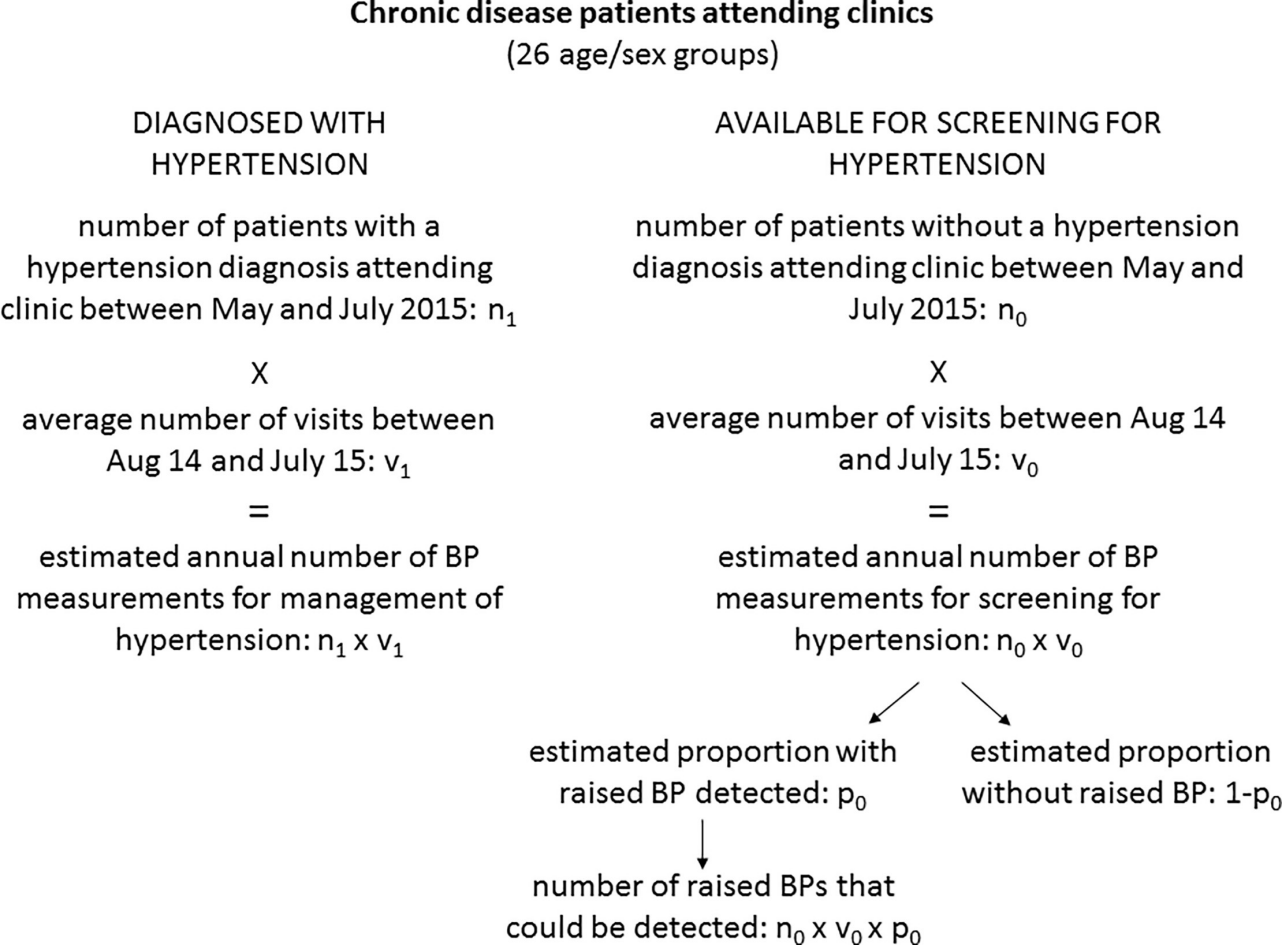
Flowchart of spreadsheet used to estimate workloads and outcomes in different scenarios.

We discarded the first three months of data collection in the clinics when the database was being built up. We collected data on chronic disease clinic activity from May 2014 to July 2015 (the end of the intervention). We found a very rapid increase in the number of routine visits for chronic diseases over these 15 months (from 3,853 visits in May 2014 to 6,726 by July 2015), partly due to the continued roll-out of antiretroviral therapy to be delivered in primary care, and partly to an on-going hospital policy of referring stable out-patients back to primary care to relieve pressures on hospital out-patient services. Because of this rapid increase in numbers, which we believe to be real and likely to be sustained, we used just the last three months of available data (May to July 2015) to estimate the number of patients attending the clinic each year (denoted as “n” in Fig 1). We calculated how many times a year each patient visited the clinic by using the average number of visits for the year August 2014 to July 2015 (denoted as “v” in Fig 1), making the assumption that although the number of patients was increasing, the frequency of visits by individual patients will remain the same. For this paper, we considered clinic patients in two categories, related to the need for screening for, or management of, hypertension:

- ‘Diagnosed with hypertension’: when the clinic file recorded a diagnosis of hypertension at any time. We treated these people as requiring regular blood pressure checks for management of their condition.
- ‘Available for screening for hypertension’: when the clinic file did not record a diagnosis of hypertension. We assumed that a proportion of these people had raised blood pressure but had not been identified and put on treatment. We derived the relevant proportion (“p”) from the findings of the two cross-sectional surveys.

### Estimating population prevalence of untreated raised blood pressure (Table 1, row 2)

Two population samples were drawn from the Agincourt Health and Demographic Surveillance System (Agincourt HDSS) population [12] in 2013 and 2015 respectively to provide estimates of population prevalence of raised blood pressure and whether respondents were taking medication for hypertension before and after the intervention. The questionnaire included questions about treatment for hypertension and other cardiovascular risk factors. People who consented to take part in either of the two surveys completed an interview in their homes. They also had their blood pressure measured three times, two minutes apart, in a seated position using the OMRON M6W automated cuff (Omron, Kyoto, Japan)[9]. We discarded the first blood pressure reading and took the mean of the second and third readings to determine a person’s systolic and diastolic blood pressure. The results of the trial showed no difference in population blood pressure levels before and after the intervention[10] [13] therefore, for this paper, we combined data from the two surveys. For the people who had their blood pressure measured in both surveys, we took the measure from the first survey. For the calculations reported in this paper we allocated participants to one of three groups:

- ‘Hypertension not on treatment’: those with a systolic pressure equal to or above 140 mmHg or a diastolic pressure equal to or above 90 mmHg and not taking any medication for hypertension.
- ‘Hypertension on treatment’: those who were taking any medication for hypertension including those with normal blood pressure on treatment.
- ‘Normal blood pressure’: those with a systolic pressure below 140 mmHg and a diastolic pressure below 90 mmHg and not on treatment.

We calculated the prevalence of the first of these three categories for each five year age and sex group (denoted by “p” in Fig 1).

### Estimating the yield of new diagnoses of hypertension following screening (Table 1, row 3)

The lay health workers in the four intervention clinics were trained to identify people found with raised blood pressure, but with no previous diagnosis of hypertension, when the routine clinic blood pressure measurements were done at the vital signs station. Such people were asked to return for a second measurement to check whether the high blood pressure was consistent. The lay health workers kept records of the people they identified in this way and we were able to identify from the clinic records how many of them returned for a second blood pressure check, and, of those who returned how many were then given a diagnosis of hypertension.

We used these data collected by the lay health workers and recorded by the clinics’ data clerks to estimate the proportion of people who are found to have a raised blood pressure who will return for a second measurement and the proportion of those who will be given a diagnosis of hypertension.

### Estimating clinic workloads and outcomes

We constructed a spreadsheet (Fig 1) to estimate likely clinic activity in different scenarios. Because the prevalence of hypertension and frequency of clinic visits are both strongly correlated with age and sex, we made all the calculations separately for men and women and by 5-year age groups and then summed the results to provide the overall total. The left hand side of the figure shows how the workload from measuring blood pressure in people with a diagnosis of hypertension was estimated. The right hand side of the figure shows how we estimated the number of blood pressure measurements in people without a diagnosis of hypertension, and how many new diagnoses of hypertension might be made if raised blood pressures were noted and the patients concerned encouraged to return for a second blood pressure check.

In doing these calculations we made several assumptions:

- That there would be the same number of patients visiting the clinics as that observed in May to July 2015. This may mean that we have underestimated the volume of work, since we have no reason to believe that the rapid increase in the numbers of patients visiting the clinics stopped at the time we finished our data collection;
- That the age and sex profile of patients visiting the clinics would remain the same;
- That, once the workload of routine blood pressure checks was reduced, health care staff would spend time to identify people with raised blood pressure and ask them to return for a second measurement;
- That the prevalence of untreated hypertension in chronic disease patients without a diagnosis of hypertension visiting the clinic is the same as that found in our population surveys; and
- That repeated blood pressure measurements in the same year are unlikely to identify any significant numbers of new cases of hypertension after the first measurement that year.

### Sensitivity analyses

We carried out two sensitivity analyses to test the robustness of our findings. First, we used data for the entire year from August 2014 to July 2015 rather than just the last three months, to construct the model. Secondly, because these data were collected as part of a randomised trial, we used data for May to July 2015 for just the four control clinics in the trial, to see whether the pattern of findings was the same in clinics which had not had the support of lay health workers.

### Ethics

Ethical approval was granted by the University of the Witwatersrand Human Research Ethics Committee (Medical) (reference M130347, M130754, M130964), the University of Warwick Biomedical and Scientific Research Ethics Committee (reference REGO-2013-062, REGO-2013-203, REGO-2013-562), and the Mpumalanga’s Provincial Research and Ethics Committee (dates of letters: 7 June 2013, 18 September 2013, 6 November 2013). The clinic manager of all the clinics taking part in the trial consented for their clinic to participate. All individuals interviewed in the population survey gave written informed consent to the interview. All clinic patients whose details were entered in the clinic database gave written informed consent.

## Results

### Clinic activity related to management of, and screening for, hypertension

Data collected in the clinics show that 8,947 patients attended one of the eight clinics for chronic disease management between May and July 2015, with a total of 22,323 visits over the three months. Nearly half, 45% (4,053) of these clinic users had a diagnosis of hypertension; these patients were markedly older than the other patients (Table 2). The vast majority of patients without a diagnosis of hypertension were attending the clinics for anti-retroviral therapy. Patients with hypertension attended clinics more frequently (overall mean of 7.1 visits in a year) compared to those without hypertension (mean 5.4 visits). During May to July 2015, 35% of visits were from patients with a diagnosis of HIV but not hypertension; 51% were from patients with a diagnosis of hypertension but not HIV; 9% of visits were from patients who had a diagnosis of both HIV and hypertension, while 5% of visits were from patients who had neither diagnosis. This latter group had a variety of diagnoses such as epilepsy, mental illness, chronic obstructive pulmonary disease and diabetes.

**Table 2:** Patients and clinic visits for chronic care: May—July 2015.

	Chronic care patients with a diagnosis of hypertension					Chronic care patients without a diagnosis of hypertension			
	Patients			Visits	Visits / patient	Patients		Visits	Visits / patient
	n		(%)	n	mean	n	(%)	n	mean
**Women**									
Age 18–39	255		(6.3)	747	2.93	2,222	(45.4)	4,058	1.83
Age 40–59	1,237		(30.5)	4,026	3.25	1,145	(23.4)	2,058	1.80
Age 60 plus	1,710		(42.2)	5,713	3.34	217	(4.4)	399	1.84
**Men**									
Age 18–39	55		(1.3)	179	3.25	534	(10.9)	1,001	1.87
Age 40–59	263		(6.5)	918	3.49	628	(12.8)	1,218	1.94
Age 60 plus	533		(13.2)	1,737	3.26	148	(3.0)	269	1.82

### Prevalence of untreated hypertension in the population

There were 8,957 individuals sampled for the two surveys. We discarded the second survey result for those 591 who were sampled twice, leaving 8,366 individuals in the sample. Of these, 6,641 (79%) individuals completed the questionnaire and are included in this analysis. Table 3 shows the prevalence of normal blood pressure, and treated and untreated hypertension by age and sex. Overall, 46.3% were found to have a raised blood pressure; and the majority (58.4%) of them were not on any treatment. In the youngest age group, a large majority had a normal blood pressure, butin those women and men who had a raised blood pressure, only a very small proportion was receiving treatment. In the oldest age group, the majority of the population had a raised blood pressure and while more of those with a raised blood pressure were on treatment, still nearly half (49%) of them were not on any treatment (Table 3).

**Table 3:** Prevalence of raised blood pressure, hypertension on treatment, and normal blood pressure from two population surveys.

Age/sex group	Raised BP*, no treatment		Hypertension on treatment		Normal blood pressure		Total	
	n	(%)	n	(%)	n	(%)	n	(%)
**Women**								
Age 18 to 39	119	(12.2)	23	(2.36)	831	(85.4)	973	(100)
Age 40 to 59	317	(26.2)	234	(19.3)	659	(54.5)	1210	(100)
Age 60 plus	545	(30.9)	682	(38.6)	539	(30.5)	1766	(100)
**Men**								
Age 18 to 39	192	(24.5)	12	(1.53)	581	(74.0)	785	(100)
Age 40 to 59	287	(32.0)	87	(9.69)	524	(58.4)	898	(100)
Age 60 plus	338	(33.5)	242	(24.0)	429	(42.5)	1009	(100)

*either diastolic BP >90mm/Hg or systolic BP > 140 mm/Hg

### Patient journey for patients found with raised blood pressure

There were 789 patients who were identified by the lay health workers in the intervention clinics as having a raised blood pressure, but not a current diagnosis of hypertension. Most of them (around 85%) were patients visiting the clinic for an acute problem; the rest had a chronic condition other than hypertension. Of the 789 identified, 23 had no age recorded and six were aged less than 18 years, leaving 760 patients. Of these, 394 (51.8%) returned within 3 months for a second blood pressure check, and of those who returned, 301 (76.4%) were given a diagnosis of hypertension (Table 4).

**Table 4:** Clinic patients with raised blood pressure identified by lay health workers, number returning for second blood pressure measurement and number diagnosed with hypertension. Data from individual clinic records.

Column	A		B		C	
	Patients with raised BP identified by LHWs		Of those identified, who returned for 2nd BP within 3 months		Of those who returned, who were diagnosed with hypertension	
	n	%	n	(% of column A)	n	(% of column B)
**Women**						
Age 18–39	159	(20.9)	70	(44.0)	38	(54.3)
Age 40–59	261	(34.3)	140	(53.6)	113	(80.7)
Age 60 plus	134	(17.6)	87	(64.9)	76	(87.4)
**Men**						
Age 18–39	46	(6.05)	11	(23.9)	9	(81.8)
Age 40–59	81	(10.7)	40	(49.4)	29	(72.5)
Age 60 plus	79	(10.4))	46	(58.2)	36	(78.3)
**Total**	760	(100)	394	(52.6)	301	(76.4)

### Managing hypertension in patients with an existing diagnosis

In the year August 2014 to May 2015, patients diagnosed with hypertension attended a clinic, on average 7.1 times (approximately every seven weeks) and had their blood pressure measured every time, creating a workload for primary care staff of 28,736 blood pressure checks (Table 5). However, South African guidelines for the management of hypertension recommend that “*once a stable target BP has been achieved*, *follow-up BP measurement should be performed every 3–6 months*” (page 79) [14]. If patients with hypertension were to be managed according to these guidelines and had their blood pressure measured every three months, nearly half (43.6%) of the workload could be eliminated (Table 5).

**Table 5:** For patients with hypertension: Change in disease management workload if blood pressure measured four times a year.

Scenario		Number of blood pressure measurements	Percentage reduction compared with first scenario
1	Current situation, all hypertension patients measured at every visit	28,736	____
2	All hypertension patients measured every three months	16,212	43.6%

### Screening for hypertension in chronic disease patients with no diagnosis of hypertension

We considered different scenarios which would relieve the pressures caused by the large numbers of blood pressure checks, while maintaining systematic screening for hypertension. Our hypothesis is that if fewer blood pressure checks were carried out then there is a greater likelihood of a raised blood pressure being noted and acted upon.

Using the numbers of patients seen in the clinics during May to June 2015, we calculated the effect of screening for hypertension once a year in those without symptoms and without diagnosed hypertension. Because of the HIV epidemic, there are large numbers of younger patients attending the clinics; 13% of women and 7% of men attending with a chronic condition were aged between 18 and 30 years, We therefore considered some possible age cut-offs for screening. Table 6 shows the outcome of different scenarios for managing blood pressure checks in chronic disease patients without a diagnosis of hypertension.

**Table 6:** Outcomes of different screening scenarios for patients without a diagnosis of hypertension.

Scenario		Number of BP measures used in screening	Number of new cases of hypertension detected	Percentage reduction BP measurements compared to scenario 1	Percentage reduction of new diagnoses of hypertension compared to scenario 1
1	Screened at every visit & newly raised blood pressure noted	27,356	418*	____	____
2	Screened all ages once a year	5,456	418	80.1%	0%
3	Patients aged 30 years and over screened once a year	4414	402	83.9% (*Or 19*.*1% of scenario 2)*	3.8%
4	Patients aged 35 years and over screened once a year	3484	371	87.3% *(Or 21*.*1% of scenario 2)*	11.2%

*we have no data on how many patients with a raised blood pressure are followed up in clinics without a LHW, but our observations suggest that it is very few.

On the basis of our observation in eight clinics from May to July 2015, we estimate that the current system will result in 27,356 blood pressure checks being required in the eight clinics every year. If appropriate action was taken on all those with a raised blood pressure, that is, patients are asked to return for a second blood pressure check and then diagnosed if raised, this would result in 418 new diagnoses of hypertension. If those chronic disease patients without a diagnosis of hypertension had their blood pressure checked only once a year, then the number of checks would be reduced by more than 80% to 5,456, without any reduction in the number of new diagnoses (Table 6).

Hypertension has a lower prevalence in younger people (Table 3), so we also considered what would be the advantages and disadvantages of limiting blood pressure screening by age and considered two scenarios: not measuring blood pressure in people aged under 30 years, and in people aged under 35 years. If the screening was limited to those over 30 years then the screening burden would be reduced by a further 3.8%, however, 3.8% of patients with raised blood pressure would not be diagnosed (Table 6). If there was a further restriction to age 35 years, there would be a further reduction of 3.4% in the workload, but at a cost of missing 11.2% of the patients with raised blood pressures (Table 6). These younger people with raised blood pressure are more likely than older people not to be on any treatment (Table 3).

### Sensitivity analyses

Table 7 shows the results of the two sensitivity analyses we carried out. These analyses are described above. We found a similar pattern of results to those reported in Table 6. Approximately 80% of the workload can be reduced if blood pressure measures are only done once a year, with no reduction in new cases detected.

**Table 7:** Sensitivity analyses results.

Scenario		Number of BP measures used in screening	Number of new cases of hypertension detected	Percentage reduction BP measurements compared to scenario 1
Sensitivity analysis using data for one year (Aug 14 to Jul 15)				
1	BP measured at every visit	32,508	482	
2	BP measured once a year	6,523	482	80%
Sensitivity analysis using data from only four control clinics May 15 to Jul 15				
1	BP measured at every visit	11,654	172	
2	BP measured once a year	2,327	172	80%

## Discussion

South Africa’s primary care clinics have been facing an unmanageable workload for some time [15]. The numbers of chronic disease patients is increasing rapidly, presenting an enormous challenge for health care workers in managing the demand appropriately. The South African Government has responded to the challenge by instituting the Integrated Chronic Disease Management Initiative, [7] which aims to improve care for chronic disease patients by, among other improvements, reducing the long waiting times in clinics. However, earlier guidance for clinics that recommended that all patients attending clinics undergo health checks to “routinely check and record weight, blood pressure, pulse and temperature” on every visit [8], has not been revisited, resulting in long queues for these checks.

During the process evaluation of a randomised control trial in eight rural primary care clinics, we observed very long queues of patients waiting to have their blood pressure checked. Patients would arrive long before the clinic opened to get an early position in the queue. There could be as many as over 100 patients in the queue. Chronic disease patients with hypertension using the control clinics in the trial reported having been in clinic for an average 3 hours and 20 minutes [11]. The long queues led to interpersonal friction at several levels. Patients argued about their position in the queue, patients complained vociferously to clinic staff about the long waits, and clinic staff argued among themselves about who should attend to the queue; a job which usually went to the most junior person [11]. Moreover, the electronic blood pressure machines supplied to clinics as part of the new initiative were frequently breaking down due to the heavy use, and the cuffs wore out quickly and became leaky. Because the machines frequently malfunctioned, there was a tendency for nurses to ignore the readings given, assuming high readings were just an error of the machine[11].

In this paper, we have used some of the data we collected to construct a spreadsheet which showed first, estimates of the workload that could be reduced by a change in policy on screening for hypertension and the frequency of checks to manage diagnosed hypertension. Secondly, we have estimated the number of new diagnoses of hypertension that might be made if staff were to respond appropriately to high readings observed in patients without a diagnosis of hypertension, and if the blood pressure machines were reliable. Reducing the number of patients each day who must have their blood pressure measured would shorten the queues and reduce waiting times. It would also reduce the wear and tear on the blood pressure machines, although a system for calibration and maintenance of the machines would still be needed.

Although our spreadsheet results also showed that an extra reduction in workload could be achieved by limiting the age range to be screened, this would be achieved at the expense of missing around 4% of all the cases on undiagnosed hypertension, and arguably some of those it is most important to treat since they have already developed hypertension at such a young age.

Even though the model used for the estimates was not sophisticated, it shows very large effects of a potential change in policy that are unlikely to be changed by more sophisticated modelling. Because we had access to electronic records of each patient’s clinic attendances we were able to give a much clearer picture of activity in a rural primary care clinic than had previously been possible. However, we only collected data from patients attending the clinics for management of chronic diseases and cannot include the workload generated by patients visiting for other reasons.

Although our model points to the benefits of a change in policy, such a change would require some investment of resources for the new system to work well. The enrolled or staff nurses who take the blood pressure measurements at the vital signs stations would have to actively consider whose blood pressure to screen, rather than just screening everybody. This would require staff training. Also, the blood pressure machines would need to be serviced regularly to ensure reliable readings. The staff nurses would need to take note of raised blood pressures, and ensure that patients were followed up for a second assessment and enrolled in care when necessary.

Hypertension represents a major health burden throughout South Africa. We have found that 60% of all visits to primary care clinics in the research area for chronic disease management were from people with a diagnosis of hypertension, while our two cross-sectional surveys showed the very high prevalence of hypertension (46.3%) in the adult population (age 18 plus). This accords with national data. The World Health Organization’s study on Global Ageing and Adult Health (SAGE) assessed the prevalence of hypertension in people aged over 50 years in six countries (China, Ghana, India Mexico, the Russian Federation, and South Africa) between 2007 and 2010. South Africa had the highest prevalence of all countries, at 77.9% (95% CI 76.4–79.4). Moreover, only 38.0% (36.2, 39.8) of the South African respondents were aware of their diagnosis [4]. Other researchers have estimated the burden of mortality attributable to high blood pressure in South Africa in 2000 [16]. Using measures of blood pressure from the 1998 South African Demographic and Health Survey, they estimated that high blood pressure had caused 9% (95% uncertainty interval 8.6–9.3) of all deaths in people aged over 30 in 2000. The authors commented that there is ‘considerable potential for health gain from implementing BP-lowering interventions’ (p 692).

The challenge of managing chronic diseases, and particularly hypertension and HIV, within primary care services is not unique to South Africa. Hypertension is increasing in prevalence throughout sub-Saharan Africa[17], and some other sub-Saharan countries also have a high prevalence of people with HIV, who, with the roll-out of anti-retroviral treatment, will require long-term management [1, 5]. Reconfiguring health services to manage large numbers of people attending routinely for the management of chronic disease is likely to become a priority throughout sub-Saharan Africa. While there is little evidence from low and middle income settings to support initiating population screening for hypertension[18], it has previously been argued that screening patients systematically when they interact with formal medical settings may be an effective policy [19] and it is this model of screening that we have considered in this paper.

As the numbers of chronic disease patients continues to rise, a review of the current policy for measuring blood pressure in primary care clinics becomes increasingly urgent. This paper provides data that can assist policy makers undertaking such a review, showing the likely effect of some alternative strategies.
